# Supplementary material for: Modeling glioblastoma heterogeneity as a dynamic network of cell states
Source: Mol Syst Biol. 2021 Sep 16;17(9):e10105. doi: 10.15252/msb.202010105 (PMC8444284; doi:10.15252/msb.202010105)
Supplement: Supplementary file 6 — Source Data for Figure 5 [file MSB-17-e10105-s004.zip › Figure5A_sourcedata/GSEA_3071/hallmarks_stateII.GseaPreranked.1623742536692/HALLMARK_IL6_JAK_STAT3_SIGNALING.html]

Details for gene set HALLMARK\_IL6\_JAK\_STAT3\_SIGNALING[GSEA]

|  || Dataset | state23071 |
| Phenotype | NoPhenotypeAvailable |
| Upregulated in class | na\_neg |
| GeneSet | HALLMARK\_IL6\_JAK\_STAT3\_SIGNALING |
| Enrichment Score (ES) | -0.2932135 |
| Normalized Enrichment Score (NES) | -0.9951801 |
| Nominal p-value | 0.4849624 |
| FDR q-value | 0.756237 |
| FWER p-Value | 1.0 |
Table: GSEA Results Summary

  

Fig 1: Enrichment plot: HALLMARK\_IL6\_JAK\_STAT3\_SIGNALING      
 Profile of the Running ES Score & Positions of GeneSet Members on the Rank Ordered List

  

| PROBE | GENE SYMBOL | GENE\_TITLE | RANK IN GENE LIST | RANK METRIC SCORE | RUNNING ES | CORE ENRICHMENT || 1 | PIM1 |  |  | 184 | 0.896 | 0.0653 | No |
| 2 | SOCS1 |  |  | 593 | 0.581 | 0.0370 | No |
| 3 | TNFRSF12A |  |  | 629 | 0.566 | 0.0981 | No |
| 4 | IRF1 |  |  | 1173 | 0.403 | 0.0147 | No |
| 5 | PTPN2 |  |  | 1319 | 0.375 | 0.0254 | No |
| 6 | ITGB3 |  |  | 1325 | 0.374 | 0.0703 | No |
| 7 | JUN |  |  | 1725 | 0.307 | 0.0104 | No |
| 8 | IRF9 |  |  | 2901 | -0.356 | -0.2335 | No |
| 9 | TNFRSF1A |  |  | 3146 | -0.413 | -0.2424 | Yes |
| 10 | CNTFR |  |  | 3176 | -0.422 | -0.1975 | Yes |
| 11 | STAT3 |  |  | 3488 | -0.537 | -0.2076 | Yes |
| 12 | STAT2 |  |  | 3496 | -0.539 | -0.1429 | Yes |
| 13 | CD9 |  |  | 3567 | -0.581 | -0.0885 | Yes |
| 14 | TNFRSF21 |  |  | 3669 | -0.660 | -0.0319 | Yes |
| 15 | A2M |  |  | 3967 | -1.110 | 0.0321 | Yes |
Table: GSEA details [plain text format]

  

Fig 2: HALLMARK\_IL6\_JAK\_STAT3\_SIGNALING: Random ES distribution      
 Gene set null distribution of ES for **HALLMARK\_IL6\_JAK\_STAT3\_SIGNALING**
